# High-throughput sequencing reveals miRNA effects on the primary and secondary production properties in long-term subcultured *Taxus* cells

**DOI:** 10.3389/fpls.2015.00604

**Published:** 2015-08-06

**Authors:** Meng Zhang, Yanshan Dong, Lin Nie, Mingbo Lu, Chunhua Fu, Longjiang Yu

**Affiliations:** ^1^Department of Biotechnology, Institute of Resource Biology and Biotechnology, College of Life Science and Technology, Huazhong University of Science and TechnologyWuhan, China; ^2^Key Laboratory of Molecular Biophysics Ministry of Education, College of Life Science and Technology, Huazhong University of Science and TechnologyWuhan, China

**Keywords:** plant-cell culture, long-term subculture, miRNA regulation, production properties conversion, *Taxus chinensis*

## Abstract

Plant-cell culture technology is a promising alternative for production of high-value secondary metabolites but is limited by the decreased metabolite production after long-term subculture. The goal of this study was to determine the effects of miRNAs on altered gene expression profiles during long-term subculture. Two *Taxus* cell lines, CA (subcultured for 10 years) and NA (subcultured for 6 months), were high-throughput sequenced at the mRNA and miRNA levels^1^. A total of 265 known (78.87% of 336) and 221 novel (79.78% of 277) miRNAs were differentially expressed. Furthermore, 67.17% of the known differentially expressed (DE) miRNAs (178) and 60.63% of the novel DE-miRNAs (134) were upregulated in NA. A total of 275 inverse-related miRNA/mRNA modules were identified by target prediction analysis. Functional annotation of the targets revealed that the high-ranking miRNA targets were those implicated in primary metabolism and abiotic or biotic signal transduction. For example, various genes for starch metabolism and oxidative phosphorylation were inversely related to the miRNA levels, thereby indicating that miRNAs have important roles in these pathways. Interestingly, only a few genes for secondary metabolism were inversely related to miRNA, thereby indicating that factors other than miRNA are present in the regulatory system. Moreover, miR8154 and miR5298b were upregulated miRNAs that targeted a mass of DE genes. The overexpression of these miRNAs in CA increased the genes of taxol, phenylpropanoid, and flavonoid biosynthesis, thereby suggesting their function as crucial factors that regulate the entire metabolic network during long-term subculture. Our current studies indicated that a positive conversion of production properties from secondary metabolism to primary metabolism occurred in long-term subcultured cells. miRNAs are important regulators in the upregulation of primary metabolism.

## Introduction

Most of the valuable natural products from plants are present at very low concentrations. For instance, only 1 kg of taxol is present in a thousand century-old *Taxus* trees. Direct isolation of these secondary metabolites from plant tissues was a rough way leading to an extinct exploration. Plant cell culture technology is a promising alternative for production of high-value secondary metabolites (Lila, 2005; Zhao and Verpoorte, 2007). However, several studies reported that a series of changes occurred in long-term subcultured cells, such that production became lower (Kolewe et al., 2008; Mustafa et al., 2011; Li et al., 2013). For instance, the long-term subcultured cells metabolically and morphologically differ; thus, the cell aggregates are heterogeneous subpopulations and individual cells within a culture accumulated in each product (Hall and Yeoman, 1987; Naill and Roberts, 2005; Kolewe et al., 2010). Patil et al. (2013) reported that the long-term subculture of *Taxus* cells formed numerous unevenly sized aggregates, thereby blocking taxol production. Moreover, the long-term subcultured cells frequently have epigenetic modifications (e.g., DNA methylation), and their cellular ploidy levels varied after long-term subculture (Baebler et al., 2005; Miguel and Marum, 2011). These observations implied that the conversion of long-term subcultures is related to a complicated and complex regulatory network. However, none of these reports showed the different gene expression profiles in long-term subcultured cells. The previous reports could not explain the regulatory mechanisms in detail.

In plants, miRNAs are important regulators of various activities, such as genome stability, development, and abiotic or biotic stress response (Jones-Rhoades and Bartel, 2004; Sunkar and Zhu, 2004; Lu et al., 2005; Mallory and Vaucheret, 2006; Vaucheret, 2006; Bartel, 2009; Xu et al., 2010). To date, several reports have combined mRNA-seq and miRNA-seq to elucidate the miRNA functions in complex problems in plants (Chen et al., 2012; Yang et al., 2012). He et al. (2013) determined the pathways involved in the rapid growth of developing culms in *Moso bamboo*. Pei et al. (2013) analyzed the miRNA and mRNA profiles in response to ethylene in rose petals during flower opening. Reynoso et al. (2013) revealed that polyribosomes selectively recruit mRNAs and miRNAs to respond to rhizobium infection in *Medicago truncatula*, thereby indicating the mechanism of nodule conformation. Therefore, the integrated analysis of miRNA and mRNA profiles could help us better understand the long-term subculture of cells.

*Taxus* sp. produces taxol, which is a widely used anticancer drug, but the taxol content is extremely low in plants (Howat et al., 2014). Given the problems of long-term subculture, current cell culture systems of paclitaxel production were not applicable for commercial use (Malik et al., 2011). Subcultured *Taxus* cells are a representative model for clarifying the conversion mechanism of long-term subculture. Therefore, two *Taxus* cell lines, namely, NA (newly separated and subcultured for 6 months with a high secondary metabolite biosynthesis ability) and CA (which is the control cell line being subcultured for 10 years with low secondary metabolite biosynthesis), were used to clarify the miRNA functions during the conversion of long-term subcultures.

## Results

### NA had a higher production of secondary metabolites than CA

**Figure 1:**
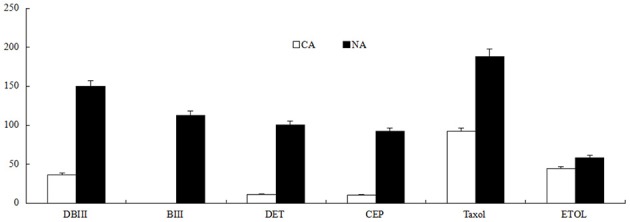
**Taxane content (μg/g DW) in CA and NA**. DBIII, 10-Deacetylbaccatin III; BIII, baccatin III; DET, 10-deacetyl taxol; CEP, cephalomanine; ETOL, 7-epi taxol.

The two cell lines, CA and NA, appeared to have different phenotypes. NA cells had slower growth and appeared bronze, whereas CA cells were beige. The secondary metabolite contents in CA and NA cell lines were significantly different. The flavonoid content in NA was 7.63 mg/g fresh weight (FW), which was 11.74 times higher than the 0.65 mg/g FW in CA. The taxane production was also quantified. Results showed that the secondary metabolite levels were significantly higher in NA than in CA (Figure 1). According to our previous report (Song et al., 2014), the production of taxane can be represented by quantifying six compounds, including taxol. Each of the six compounds had significantly higher levels in NA (Figure 1), the amount of DBIII (10-deac), DET, CEP, and taxol was 3.12, 5.50, 5.18, and 1.88 times higher, respectively, than that in CA. BIII is an important precursor in taxane biosynthesis; notably, this compound could not be detected in CA, whereas the BIII content in NA was as high as 118 μg/g DW. All these results indicated that NA had stronger biosynthesis of secondary metabolites.

### Sequencing and annotation of miRNA-seq

**Figure 2:**
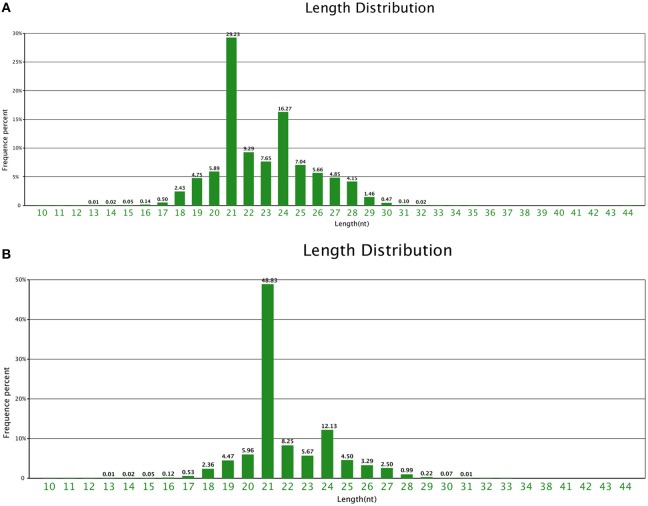
**Small RNA length distribution**. Length distribution of small RNAs in **(A)** CA and **(B)** NA.

A total of 19,998,242 and 19,686,076 raw reads were sequenced, with 19,843,361 and 19,558,835 high quality reads. Finally, 19,500,319 and 19,212,618 clean reads were generated in CA and NA, respectively (Table S1). In the two samples, 21 nt-length and 24 nt-length small RNAs were the majority, although the 21 nt-length small RNA was the most dominant in NA and CA. In particular, the 21 nt-length small RNA was 48.83% of all the small RNAs in NA, with almost 1.8 times higher redundancy than CA (Figure 2).

Further annotation analysis divided these small RNA into miRNA, rRNA, snRNA, snoRNA, tRNA, and unannotated RNA by BLAST searches with public databases. In CA, 13,527 unique sequences (0.65%) of the total 712,820 sequences (3.66%) were annotated as miRNAs; 215 of which were identified as known miRNAs. In NA, 18,008 unique sequences (0.61%) of the total 909,051 sequences (4.73%) belonged to 286 miRNAs (Table 1). A total of 351 known miRNAs were identified, and 336 (95.73%) were detected in both CA and NA.

**Table 1:** **Statistic of small RNA sequences**.

	**Unique sRNAs**		**Total sRNAs**	
	**CA**	**NA**	**CA**	**NA**
miRNA	13,527 (0.65%)	18,008 (0.61%)	712,820 (3.66%)	909,051 (4.73%)
rRNA	101,671 (4.87%)	92,394 (3.14%)	9, 493,261 (48.68%)	5, 313,742 (27.66%)
snRNA	2327 (0.11%)	1995 (0.07%)	18,534 (0.10%)	8704 (0.05%)
snoRNA	848 (0.04%)	955 (0.03%)	3716 (0.02%)	3410 (0.02%)
tRNA	13,319 (0.64%)	12,805 (0.43%)	460,460 (2.36%)	562,054 (2.93%)
Unann	1, 955,441 (93.69%)	2, 820,464 (95.72%)	8, 811,528 (45.19%)	12, 415,657 (64.62%)
Total	2, 087,133 (100%)	2, 946,621 (100%)	19, 500,319 (100%)	19, 212,618 (100%)

**Figure 3:**
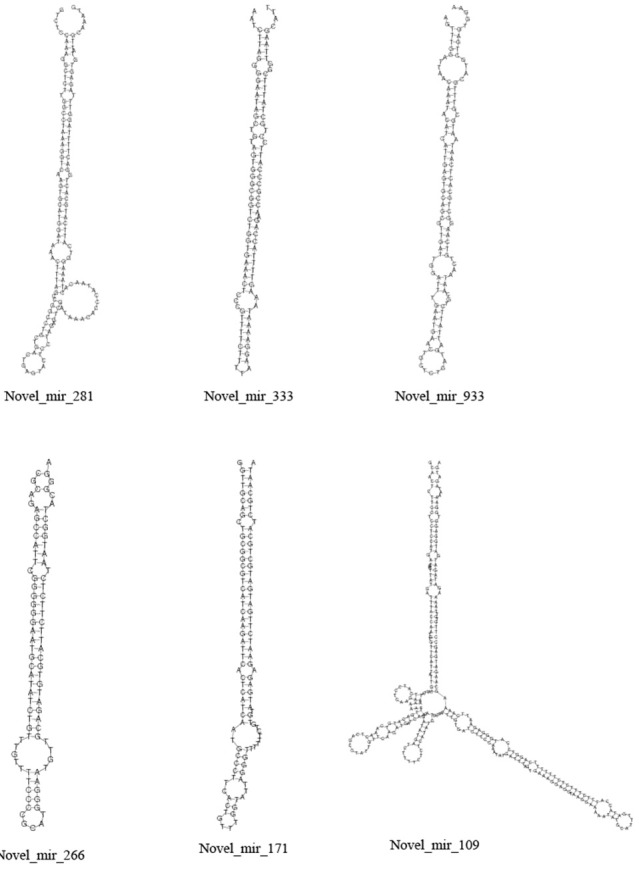
**Stem–loop structure of selected novel miRNAs**.

The unannotated sRNAs were predicted with strict conditions to find novel miRNAs. After alignment with the simplified genome of *Taxus baccata* (Nystedt et al., 2013), only the miRNAs whose precursors could form a hairpin structure were considered to be miRNAs. Otherwise, these sRNAs were designated as pseudo-miRNAs. A total of 482 and 575 novel miRNAs were predicted in CA and NA, respectively. Only 277 novel miRNAs were detected in both CA and NA. Several variant structures of novel miRNAs are shown in Figure 3.

### Most miRNAs changed their expression levels

**Figure 4:**
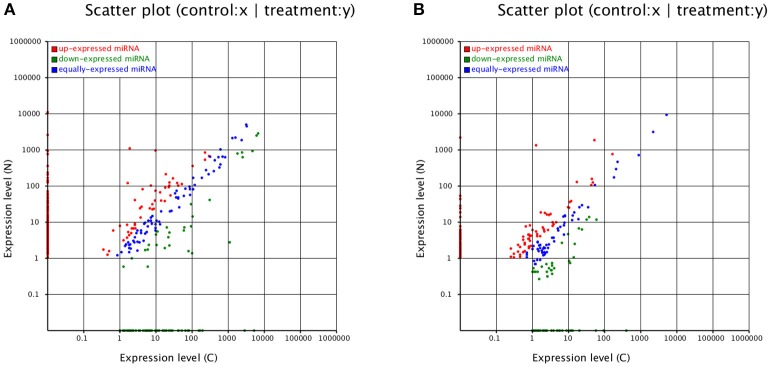
**Expression levels of miRNAs**. Expression levels of **(A)** known miRNAs and **(B)** novel miRNAs.

According to our data, most miRNAs changed their expression levels (Figure 4). Among the 336 known miRNAs in CA, miR2199, miR2916, miR397a, and miR164c were the most overrepresented, whereas novel_mir_281 and novel_mir_333 were the most overrepresented of the 277 novel miRNAs (Table S2). miR397 expression was significantly higher in NA, which was hardly detected with 0.01 TPM (transcript per million) in CA. Moreover, miR2199 and miR2916 were still highly abundant in NA. The novel miRNAs novel_mir_281 and novel_mir_333 were still the most overrepresented in NA. However, the abundance of novel_mir_933, novel_mir_266, and novel_mir_171 became the highest in NA, although all three were hardly detected in CA (Table S2). Quantitative real-time PCR (qRT-PCR) was conducted, and the results agreed with the high-throughput dataset (Figure S1).

Among the 336 known miRNAs, 265 (78.87%) were differentially expressed. Only 178 miRNAs (approximately 67.17% of the 265 DE-miRNAs) were upregulated in NA. In addition, 221 miRNAs (79.78% of the 277 novel miRNAs) were differentially expressed, whereas 134 (60.63%) were upregulated in NA (Table S2). In summary, known and novel miRNAs exhibited highly diverse expression patterns, thereby indicating their important roles during long-term subculture.

### Prediction and annotation of DE-miRNA targets

**Figure 5:**
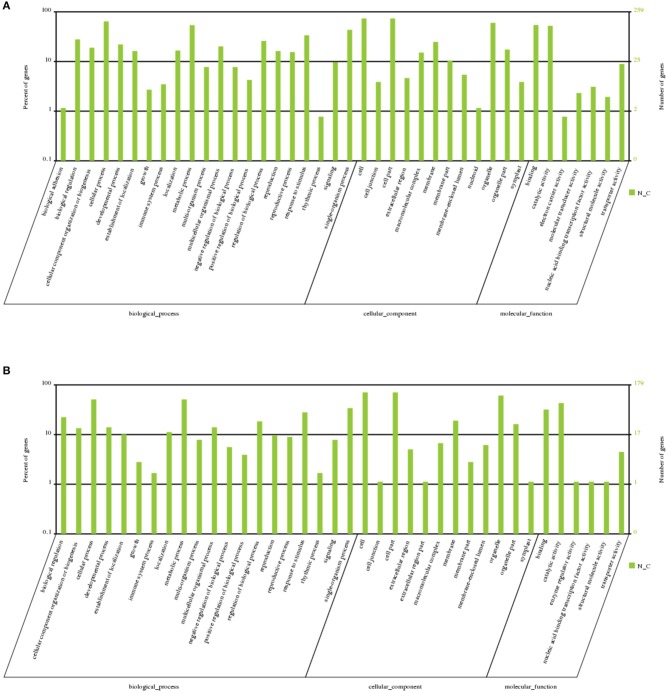
**GO analysis of the targets of DE-miRNAs**. GO analysis of **(A)** DE known miRNA targets and **(B)** DE novel miRNA targets.

To understand the possible biological functions of DE-miRNAs, mRNA-seq was simultaneously performed. Among the 265 known and 221 novel DE-miRNAs, 150 known and 137 novel miRNAs targeted 481 and 449 genes, respectively, with a total of 914 target genes (Table S3). The cleavage information is shown in Table S4. GO (Gene Ontology) enrichment analysis showed that the targets were mostly enriched in “cellular process,” “metabolic process,” “cell part,” “binding,” and “catalytic activity” (Figure 5). Further GO enrichment multiple analysis showed that “energy coupled proton transmembrane transport, against electrochemical gradient” (GO: 001598814), “ATP hydrolysis coupled proton transport” (GO: 0015991), “baruol synthase activity” (GO: 0080011), “thioglucosidase activity” (GO: 0019137), and “transcription factor TFIIE complex” (GO: 0005673) were the enriched GO terms, thereby indicating that known DE-miRNAs regulated these processes during long-term subculture. Meanwhile, “detoxification of nitrogen compound” (GO: 0051410), “response to nitrosative stress” (GO: 0051409), “response to L-ascorbic acid” (GO: 0033591), “cyanoalanine nitrilase activity” (GO: 0047427), “indole-3-acetonitrile nitrile hydratase activity” (GO: 0080109), “indole-3-acetonitrile nitrilase activity” (GO: 0080061), and “3-cyanoalanine hydratase activity” (GO: 0047558) were the most enriched terms of novel DE-miRNAs (Figure S2).

The up- and down- regulated targets were separately subjected to GO analysis. According to our results, several pathways were increased in CA but were not found in NA, such as the pathways for antioxidants, electron carriers, biological adhesion, and ribonucleoprotein complexes. Although the genes of these pathways account for a small percentage of the DE targets, their presence indicated that CA was more likely to have a better lifespan (Figure S3).

Only 282 and 218 genes with KEGG pathway annotation were targeted by known and novel miRNAs, respectively. Among the targets of known miRNAs, “metabolic pathways,” “biosynthesis of secondary metabolites,” “plant–pathogen interaction,” “plant hormone signal transduction,” “pyrimidine metabolism,” “purine metabolism,” “RNA transport,” and “oxidative phosphorylation” were the highly ranked pathways (Table S5). Among the targets of novel miRNAs, “metabolic pathways,” “biosynthesis of secondary metabolites,” and “protein processing in endoplasmic reticulum” were the most highly ranked. These results indicated that these pathways were the main targets of DE-miRNAs during long-term subculture.

### Analysis of DE-miRNA and their target genes

Among the 150 known DE-miRNAs with target genes, 110 were upregulated and 40 were downregulated. Among their 481 target genes, 245 genes (50.94%) were differentially expressed in NA, 117 were upregulated, and 127 were downregulated. The 110 upregulated known miRNAs targeted 390 genes, with 98 upregulated and 93 downregulated. The 40 downregulated known miRNAs targeted 104 genes, with 24 upregulated and 41 downregulated (Table S3).

Moreover, among the 137 novel miRNAs, 83 were upregulated and 54 were downregulated. For the 449 target genes, 256 (57.02%) were differentially expressed genes in NA, in which 108 were upregulated and 148 were downregulated. The 83 upregulated novel miRNAs targeted 293 genes, with 76 upregulated and 91 downregulated. The 54 downregulated novel miRNAs targeted 181 genes, with 38 upregulated and 63 downregulated.

### miRNA/mRNA modules mainly involved in primary metabolism and abiotic or biotic stress responses

#### Oxidative phosphorylation

A total of 11 downregulated genes among the 22 target genes were targeted by 8 known and 2 novel upregulated miRNAs. All miRNAs were barely expressed in CA, thereby indicating that oxidative phosphorylation was mainly regulated by miRNAs. The 11 downregulated genes included cytochrome c oxidase subunit 2, succinate dehydrogenase (ubiquinone) cytochrome b560 subunit, V-type H+-transporting ATPase subunit F, NAD(P)H-quinone oxidoreductase subunit 5, and NADH-ubiquinone oxidoreductase chains 2 and 4 (Table S6).

#### RNA polymerase

Only 13 of the 21 targeted genes were targeted by 15 DE known miRNAs (13 upregulated and 4 downregulated) and 2 upregulated novel miRNAs. Among these genes, 9 targets were differentially expressed, in which 5 were upregulated and 4 were downregulated. Ten modules were inversely related, with 4 upregulated miRNAs, namely, miR5056, miR5499, miR6230-3p, and miR7719, which downregulated two genes that coded for the DNA-directed RNA-polymerase subunit β. The downregulated miR5509 and miR7812 were inversely related to the RPABC subunit of RNA polymerases I, II, and III, as well as the maintenance of ploidy protein MOB1 (MPS1 binder 1) (Table S6).

#### Pyrimidine and purine metabolism

A total of 25 targeted genes were targeted by 22 DE known miRNAs (17 upregulated and 5 downregulated) and 7 novel miRNAs (6 upregulated and 1 downregulated). Only 15 targets were differentially expressed, in which 8 were upregulated and 7 were downregulated. Likewise, 15 modules were inversely related. For example, DNA-directed RNA polymerase, DNA polymerase, and ATP-dependent DNA helicase RecG were downregulated by the respective miRNAs (Table S6).

Additionally, several inversely related miRNA/mRNA modules were involved in RNA transport, DNA replication, ribosomes, and so on. These results indicated that miRNAs had important roles in regulating bioactivity for the supply of essential materials and energy, such as oxidative phosphorylation and RNA polymerase. Moreover, most of the miRNAs were upregulated, whereas their targets were downregulated in NA, thereby indicating that these pathways were repressed by miRNAs during long-term subculture.

#### Plant hormone signal transduction and plant–pathogen interaction

A total of 52 genes were targeted by 30 DE-miRNAs (24 upregulated and 6 downregulated). Only 30 targets were differentially expressed, with 18 upregulated and 12 downregulated. Among the 13 inversely related miRNA/mRNA modules, brassinosteroid insensitive 1-associated receptor kinase (miR5819), disease resistance protein RPS2 (miR6161d), mitogen-activated protein kinase kinase (miR7535), mitogen-activated protein kinase kinase kinase (miR2919), auxin response factor (miR7540a), pathogenesis-related protein 1 (novel_mir_109), and extracellular signal-regulated kinase (miR1044-3p) were downregulated. By contrast, transcription factor TGA (novel_mir_73) and disease resistance protein RPM1 (miR821a) were upregulated. These results indicated that the SA, brassinosteroid, and auxin signal transduction processes, as well as the plant–pathogen interaction responses, were mainly regulated by miRNAs during long-term subculture. Additionally, CA seemed to be more sensitive to abiotic or biotic stress signals (Table S6).

### miRNA/mRNA modules are involved in phenylpropanoid, flavonoid, and terpenoid biosynthesis

Phenylpropanoid, flavonoids, and terpenoid are the most dominant secondary metabolites in plants. Their miRNA regulatory mechanisms are representative models for studying secondary metabolism during long-term subculture.

Among the 32 targeted genes, 19 were targeted by 10 upregulated known miRNAs and 1 downregulated novel miRNA. A total of 12 targets were differentially expressed, in which 11 were upregulated and 1 was downregulated. Among the 14 miRNA/mRNA modules, only 2 were inversely related: miR7696a-5p/peroxidase and novel_mir_331/gibberellin 2-oxidase.

The taxol biosynthesis genes TAT, T5H, and T10H were targeted by miRNAs. miR2645, which targets TAT, was upregulated. However, its expression was not inversely related. miRNAs seem to have a limited role in the regulation of secondary metabolism.

### miR8154 and miR5298b are important regulators of long-term subculture

**Figure 6:**
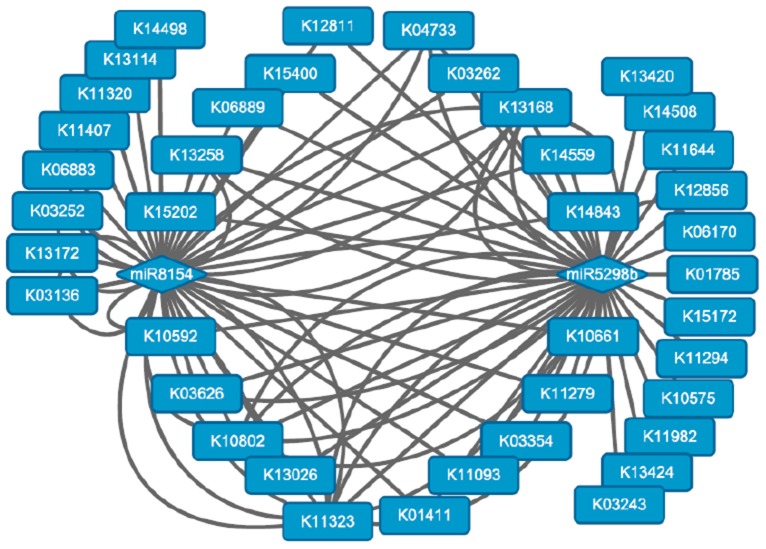
**Biological pathways regulated by miR8154 and miR5298b**. The round rectangles represented the KO IDs of the KEGG pathways.

Overall, 1059 miRNA/mRNA modules were identified, but only 275 (25.97%) were inversely related. Additionally, two known upregulated miRNAs, miR8154 and miR5298b, were identified as important regulators in the network by the Cytoscape software (download available at http://www.cytoscape.org/). These miRNAs regulated a series of genes involved in various pathways, including transcription factors, methyltransferases, and functional enzyme genes (Table S7; Figure 6). Both miRNAs were inversely related to 11 DE target genes during long-term subculture, including histone arginine demethylase, presenilin enhancer 2, ribosomal protein S21, and translation initiation factor 3. These results indicated that the methylation level, protein processing, and transcriptional level changed during long-term subculture, and these miRNAs and genes may be correlated to the conversion of subculture.

**Figure 7:**
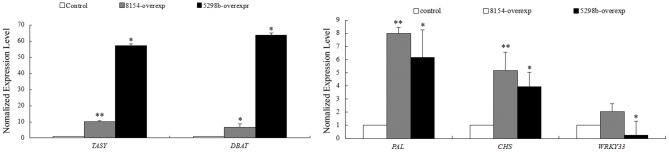
**Expression profiles of selected genes in CA cells with miR8154 and miR5298b overexpression**. *Tasy*, Taxadiene synthase; *DBAT*, 10-deacetylbaccatin III-10-O-acetyl transferaseferase; PAL, phenylalanine ammonia lyase; CHS, chalcone synthase. Error bars indicate SE. ^**^indicates *p* < 0.01; ^*^indicates *p* < 0.05.

miR8154 and miR5298b were over-expressed in CA, and the expression of the five selected genes was quantitatively detected in *Taxus* cells. WRKY33 and miR5298b followed an up–down module according to our dataset, whereas WRKY33 was downregulated by 3.99 times in the miR5298b-overexpressing lines. These results confirmed that miR8154/miR5298b regulated their targets as revealed by the high-throughput dataset. To detect whether the two miRNAs affected the secondary metabolism, the expression of *tasy* (*taxadiene synthase*), *dbat* (*10-deacetylbaccatin III-10-O-acetyl transferase*), *pal* (*phenylalanine ammonia lyase*), and *chs*, which are the respective key genes of taxol, phenylpropane, and flavonoid biosynthesis, were detected. Interestingly, all these genes were significantly upregulated in miR8154- and miR5298b-overexpressing cells (Figure 7). However, miR8154 and miR5298b could not target these genes. According to our high-throughput dataset, miR8154 and miR5298b were upregulated in NA cells. Their overexpression seemed to promote the recovery of metabolic networks in CA to their initial status, which was similar to that in NA. These results indicated that miR8154 and miR5298b had important roles in the network conversion during long-term subculture.

## Discussion

Subcultured cells were separated from cooperated organisms, with a series of significant differences after long-term subculture. For example, our work showed that long-term subcultured cells turned white from brown, and their production greatly changed in terms of composition and yield. Unlike natural bioactivity, the conversion of long-term subcultured cells was a result of multiple cellular bioactivity involving comprehensive metabolic pathways. However, previous reports focused on epigenetic variation, morphological stability, gene mutations, and so on; none of these reports studied the altered expression and regulatory systems of the long-term subcultured cells (Kiselev et al., 2011; Miguel and Marum, 2011; Smulders and De Klerk, 2011; Neelakandan and Wang, 2012; Wang and Wang, 2012; Rival et al., 2013; Kwiatkowska et al., 2014). Differences in the gene expression profiles could prominently reveal the responses of cells during adaptation to novel environments. miRNAs are known to control processes, such as development, signal transduction, response to environmental stress, and pathogen invasion, mainly at the post-transcriptional level (He and Hannon, 2004; Mahajan et al., 2011; Sun, 2012; Sunkar et al., 2012; Zeng et al., 2014). Thus, miRNA regulation has a crucial role. Consequently, two *Taxus* cell lines, CA (subcultured for 10 years) and NA (freshly separated and subcultured for 6 months), were quantified at the mRNA and miRNA levels by high-throughput sequencing in this study. Our data could promote the understanding of miRNA regulatory systems in plants and give insights into the long-term subculture of cells.

Plant miRNAs only have a small number of mRNA targets (~150), which is less than 1% of the protein-coding genes in *Arabidopsis* (Addo-Quaye et al., 2008; German et al., 2008; Li et al., 2010; Sunkar et al., 2012). Our data showed that 915 genes were targeted by known and novel miRNAs, which was approximately 2% of the coding genes; known miRNAs targeted 481 protein-coding genes (approximately 1%). These results indicated that our miRNA data had excellent coverage. Among these targets, three taxol biosynthetic enzyme genes (TAT, T10H, and T5H) were identified as miRNA targets for the first time in this study. This information provides a foundation for studying the miRNA-directed regulation of taxol biosynthesis (Qiu et al., 2009; Hao et al., 2012). Several genes were also newly targeted by miRNAs, especially by some novel miRNAs. These data could improve the current understanding of miRNA regulation.

The miRNAs regulate their targets through at least four mechanisms. Translational inhibition of the targets is the most important regulatory mechanism in animals, whereas direct cleavage of the target mRNAs is predominant in plants (Chen, 2009; Jones-Rhoades, 2012; Pei et al., 2013). Therefore, the expression of most plant miRNAs and their targets are inversely related. Several reports of miRNA regulatory systems focused on the inversely related miRNA/mRNA modules (Ori et al., 2007; Yang et al., 2012; Zeng et al., 2014). Among the 1058 DE-miRNA/mRNA modules in our dataset, only 275 modules (26.0%) were inversely related, whereas another 302 modules (28.5%) were positively related. The mRNAs of 481 modules (45.5%) had no significant differential expression. According to our data, the positively related modules had an equal number as the inversely correlated miRNA/target pairs. These results were highly similar to the work of Lopez-Gomollon et al. (2012). In addition, previous reports indicated that non-inversely related modules were functional groups (Kidner and Martienssen, 2004; Nikovics et al., 2006; Kawashima et al., 2009), but our study focused mainly on the inversely related modules.

Among the 275 inversely related modules, 68 miRNAs (39 known and 29 novel) and 114 potential targets formed 125 modules with functional annotation. A total of 101 modules (80.8%) were up–down modules, which indicated that the genes were repressed in NA, including those for the ATP-binding cassette, transcription initiation factor TFIID, E3 ubiquitin-protein ligase synoviolin, ATP-dependent DNA helicase, protein transport protein, 5′–3′ exoribonuclease, DNA mismatch repair protein, and histone arginine demethylase, among others. Moreover, most genes of the affected pathways, including oxidative phosphorylation, RNA polymerase, purine and pyrimidine metabolism, plant hormone signal transduction, and plant–pathogen interaction, were also downregulated in NA. All these results indicated that miRNAs were one of the essential regulators of primary metabolism during long-term subculture. Plant hormone signal transduction and plant–pathogen interaction are important pathways in plant defense, development, and stress responses; our results indicated that they were also mainly regulated by miRNAs.

According to the above results, several somatic mutations were related to miRNAs. First, the long-term subcultured cells grew faster than the newly derived cells because of the stronger primary metabolism. For example, stronger activity of oxidative phosphorylation could provide more energy for cell growth. Activities such as oxidative phosphorylation and DNA replication were mainly regulated by several differentially expressed miRNAs. Second, long-term subcultured cells were more likely to undergo gene mutation. In CA, several components of the mismatch recognition complex were not upregulated with the increased DNA replication. The expression of MSH6, PMS2 (downregulated), and MLH1 (unDE) indicated that the possibility of errors was higher when DNA is replicated. The functions of the miR6167/MSH6 (both upregulated in CA) and novel_mir_327/MLH1 (down-unDE modules) modules should be further clarified for gene mutations. Moreover, the targets that were annotated as multi-organism processes by GO analysis were all downregulated in CA. Therefore, these miRNAs, including miR5509, miR1312, and miR7812, among others, were related to cellular morphological changes (Table S3). Additionally, miRNAs were inversely related to several genes that encoded methylases and demethylases, thereby indicating that miRNAs probably affected the epigenetic modifications (Table S3).

By contrast, the biosynthesis of phenylpropanoid, flavonoids, and terpenoid was rarely affected by miRNA. Among the 18 modules formed by 11 miRNAs and 16 genes, which were related to these pathways, only the miR7696a-5p/peroxidase and novel_mir_331/gibberellin 2-oxidase modules were inversely related. All other relevant miRNAs were upregulated, and most of their targets were upregulated in NA, except for cinnamyl alcohol dehydrogenase, UDP-glucosyl transferase, gibberellin 3-β-dioxygenase, and naringenin 3-dioxygenase. The four genes were not significantly differentially expressed during long-term subculture. These results indicated that although miRNAs could regulate secondary metabolism, differences in the secondary metabolism were not directly affected by miRNAs during long-term subculture. Perhaps miRNAs functioned in an indirect manner to influence secondary metabolites. According to Alexandre et al. miR393 could repress auxin signaling to prevent auxin signaling from antagonizing SA signaling. Thus, miR393 re-directs secondary metabolite biosynthesis away from camalexin and toward glucosinolates (Robert-Seilaniantz et al., 2011). In our data, plant hormone signal transduction and plant–pathogen interaction were apparently regulated by miRNAs; even miR7540a could suppress auxin response factors such as miR393 in NA. Therefore, miRNAs may indirectly redirect secondary metabolite biosynthesis. Additionally, the upregulated miR8154 and miR5298b were identified to control a series of genes involved in various pathways, including those encoding transcription factors, methyltransferases, and functional enzymes. Their overexpression showed that the key genes of secondary metabolite biosynthesis were significantly upregulated, although these genes were not targeted by miR8154 and miR5298b. Interestingly, overexpression of solely miRNA was not capable to increase the secondary metabolites significantly, indicating the two miRNA were not the most original factors of the conversion. The conversion of subcultured cells were considered as the integrative results of many factors including miRNAs. Further analysis may help us understand the complex regulatory network of long-term subculture.

Overall, the secondary metabolite production of long-term subcultured cells decreased. Variations, such as epigenetic variation, morphological stability, gene mutations, and cellular ploidy levels, were considered to be the partial reason for the decreased production. Moreover, our work showed that long-term subcultured cells converted their production properties, thereby increasing primary metabolism and decreasing secondary metabolism. Given the limited production capacity of cells, conversion should be beneficial for cell growth. Therefore, conversion seems to be a positive response to novel environments, and factors such as epigenetic variation, morphological stability, gene mutations, and cellular ploidy level contributed to the positive conversion. Our results indicated that miRNAs are comprehensively involved in the conversion during long-term subculture. The primary metabolism and secondary metabolism are targets of the miRNA regulatory system. In particular, primary metabolism was effectively and principally regulated by miRNAs. In addition, secondary metabolism was not regulated by the miRNA regulatory system. The effects of the miRNA regulatory system on secondary metabolism were very faint during long-term subculture. For example, long-term subcultured cells increased their rRNA levels by 1.8 times. Interestingly, a large number of unknown 21 nt-length sRNAs was simultaneously reduced, thereby indicating that the unknown 21 nt-length sRNAs had important roles during conversion. No tissue is immune to free viral infections in plants and retroviral elements can be from the plant genome although the callus were detoxified. Therefore, considering the inserted virus genome in plants, virus-derived siRNAs may be a reason of the accumulation of unknown sRNAs in NA. Further studies about the unannotated 21 nt-length sRNAs should be conducted to clarify the novel regulation factor of the conversion during subculture. The discovery of miR8154, miR5298b, taxol biosynthetic genes, and other functional genes provides abundant information on the miRNA regulatory system and could promote our understanding of the long-term subculture of plant cells.

## Materials and methods

### Plant materials

CA was established from callus cultures derived from culture-initiated embryos, which were excised from the nascent tender stems of *Taxus chinensis* in May 2003, the callus were detoxified, and maintained with modified Gamborg's B5 medium at 25°C in the dark (Li et al., 2012). The NA cell line was established from callus cultures, which were newly derived from *T. chinensis* leaves in May 2013 and maintained with modified Gamborg's B5 medium for 6 months before RNA extraction. In addition, CA and NA were derived from the same organism, which was cultivated in the nursery of the Huazhong University of Science and Technology.

### Total RNA extraction

Cells were harvested in a microfuge tube, immersed in TRIzol Regent (Invitrogen, USA), and immediately frozen in liquid nitrogen before total RNA extraction. The E.Z.N.A.® Plant RNA Kit (Omega, USA) can effectively inhibit the RNA degradation influenced by polysaccharides and phenolic compounds in NA cells; thus, the kit was used to extract the total RNA from all samples. Total RNA of each cell line was treated with DNase I (Invitrogen) and sent to Beijing Genomics Institute-Shenzhen (Shenzhen, China) for mRNA/miRNA purification and sequencing with Illumina HiSeq2000 technology. Samples of NA and CA were mixed with RNA from three independent bio-repeats. Moreover, two other samples were sent for high-throughout sequencing to validate the results of NA and CA.

### Assembly and analysis

#### mRNA-seq

The assembly analysis of mRNA-seq was conducted according to our previous report (Li et al., 2012).

#### miRNA-seq

The 18–30 nt long small RNAs were collected. The 5′ and 3′ adaptors were added before sequencing by synthesis (SBS) with Hiseq2000 technology. The 49 nt long sequence tags were subjected to data cleaning analysis to obtain credible clean tags. The Tag2miRNA software annotated these clean tags into different categories via alignment with miRBase by taking full account of miRNA conservation. First, two mismatches and free gaps were allowed when aligning clean tags to the miRNA precursor/mature miRNA of all plants/animals in miRBase by considering the differences among species. Second, the miRNA with the highest expression was chosen for each mature miRNA family and added to a temporary miRNA database. All the annotated tags were designated as known miRNAs. For the non-annotated tags, the Mireap software was used to predict the novel miRNAs by alignment with the simplified *T. baccata* genome; the algorithm was set according to a previous report (Zhang et al., 2013). The miRNAs that could not fold into hairpin structures were regarded as pseudo-miRNAs (Nystedt et al., 2013). The known and novel miRNAs were aligned with the simplified *T. baccata* genome to verify their hairpin structure. The known miRNAs and novel miRNAs of the two samples were obtained. Subsequently, the targets of the miRNAs were predicted (Allen et al., 2005; Schwab et al., 2005), whereas GO enrichment and KEGG pathway analysis were performed on the target genes.

### Identification of differentially expressed tags

#### miRNA-seq

The expression of known miRNAs was generated by adding the number of tags that aligned to the temporary miRNA database within two mismatches. The expression of novel miRNA is produced by adding the number of miRNAs with no more than three mismatches on the 5′ and 3′ ends but with no mismatches in the middle based on the alignment result. The same method was used to identify differentially expressed miRNA for the known and novel miRNAs. First, the expression of miRNA in both samples (CA and NA) was normalized to obtain the expression in TPM: TPM = actual miRNA count/total count of clean reads × 1,000,000. Second, the fold-change was calculated and represented as log 2(TPM-NA/TPM-CA). The *P*-value of TPM for the two samples was also determined. miRNAs with a fold-change of >1 or < –1 and *p* < 0.05 (FDR ≤ 0.05) were considered to be differentially expressed and designated as DEmR. The DEmRs with *p* < 0.01 were considered to have significant differential expression.

### Vector construction and transient transformation

First, the mature miRNA and miRNA^*^ sequences of pre-miR319 were replaced by the mature miR8154 and miR5298b sequences, respectively, which constituted the artificial pre-miR8154 and pre-miR5298b. The artificial sequences were cloned into pBI121 to replace the *GUS* gene and construct the respective overexpression vectors. The transient transformation was conducted as described in a previous report (Li et al., 2012). Changes in the expression of both miRNAs were verified by qRT-PCR in transient cells.

### qRT-PCR

For the qRT-PCR of mRNAs, 1 mg of the DNase I-treated total RNA was used to synthesize cDNA with M-MLV (Promega) and poly(dT)_18_ oligonucleotides. The U6 snRNA was obtained by a BLAST search of the *T. baccata* genome sequences with the U6 sequence in *Arabidopsis thaliana*; this snRNA was used as the reference gene (Nystedt et al., 2013). SYBR Green PCR Master Mix (Applied Biosystems) was used in all qRT-PCR experiments. Changes in the relative expression of miRNAs and genes were calculated via the 2ΔΔC_t_ method. Primers used in all the qRT-PCR experiments are listed in Table S8.

## Author contributions

Conceived and designed the experiments: CF, MZ, and LY. Performed the experiments: YD, MZ, and LN. Analyzed the data: MZ and CF. Contributed reagents/materials/analysis tools: LY, CF, ML. Wrote the paper: MZ, CF, and LY.

### Conflict of interest statement

The authors declare that the research was conducted in the absence of any commercial or financial relationships that could be construed as a potential conflict of interest.

## Supplementary material

Figure S1 **qRT-PCR of several selected miRNAs**.

Figure S2 **Enrichment analysis of miRNA targets**.

Figure S3 **GO analysis of up- and downregulated targets**.

Table S1 **Quality statistics of sequencing tags of miRNAs**.

Table S2 **Expression profiles of miRNAs**.

Table S3 **Targets of miRNAs**.

Table S4 **Target and cleavage sites of miRNAs**.

Table S5 **KEGG analysis of miRNA targets**.

Table S6 **miRNA/target modules involved in several pathways**.

Table S7 **Targets of miR8154 and miR5298b**.

Table S8 **Primers used in qRT-PCR**.

## Abbreviations

DE: Differentially expressed
FW: fresh weight
DW: drought weight
DBIII: 10-Deacetylbaccatin III
DET: 10-deacetyl taxol
CEP: cephalomanine
ETOL: 7-epi taxol
BIII: baccatin III
qRT-PCR: quantitative Real Time-PCR
Tasy: taxadiene synthase
Dbat: 10-deacetylbaccatin III-10-O-acetyl transferaseferase
Pal: phenylalanine ammonia-lyase
Chs: chalcone synthase.
