# Supplementary figures and images for: High-throughput sequencing reveals miRNA effects on the primary and secondary production properties in long-term subcultured Taxus cells
Source: Front Plant Sci. 2015 Aug 6;6:604. doi: 10.3389/fpls.2015.00604 (PMC4527571; doi:10.3389/fpls.2015.00604)

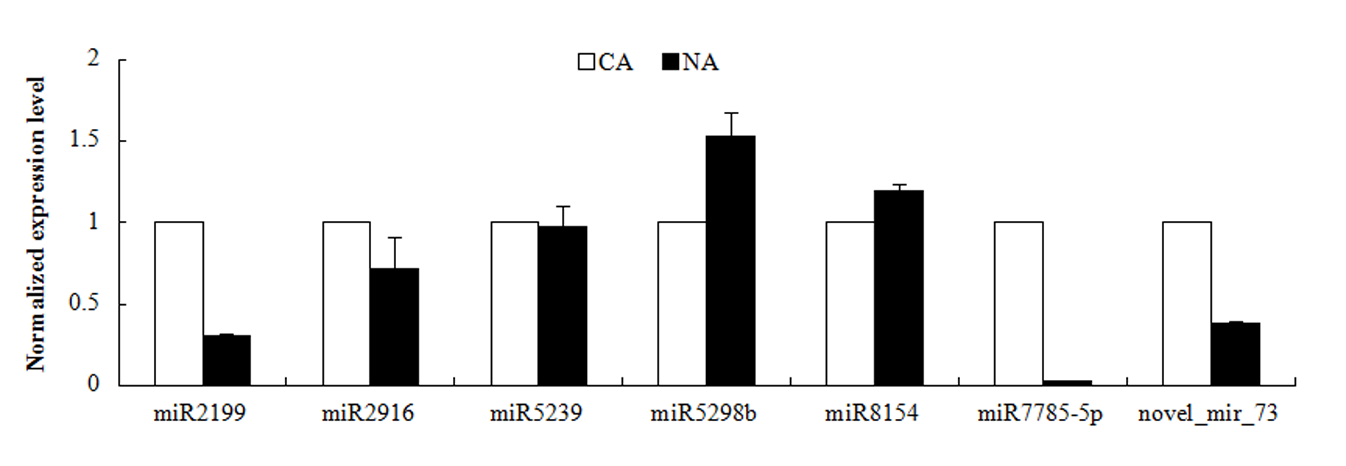

Supplement: Figure S1 — qRT-PCR of several selected miRNAs. [file Image1.TIF]

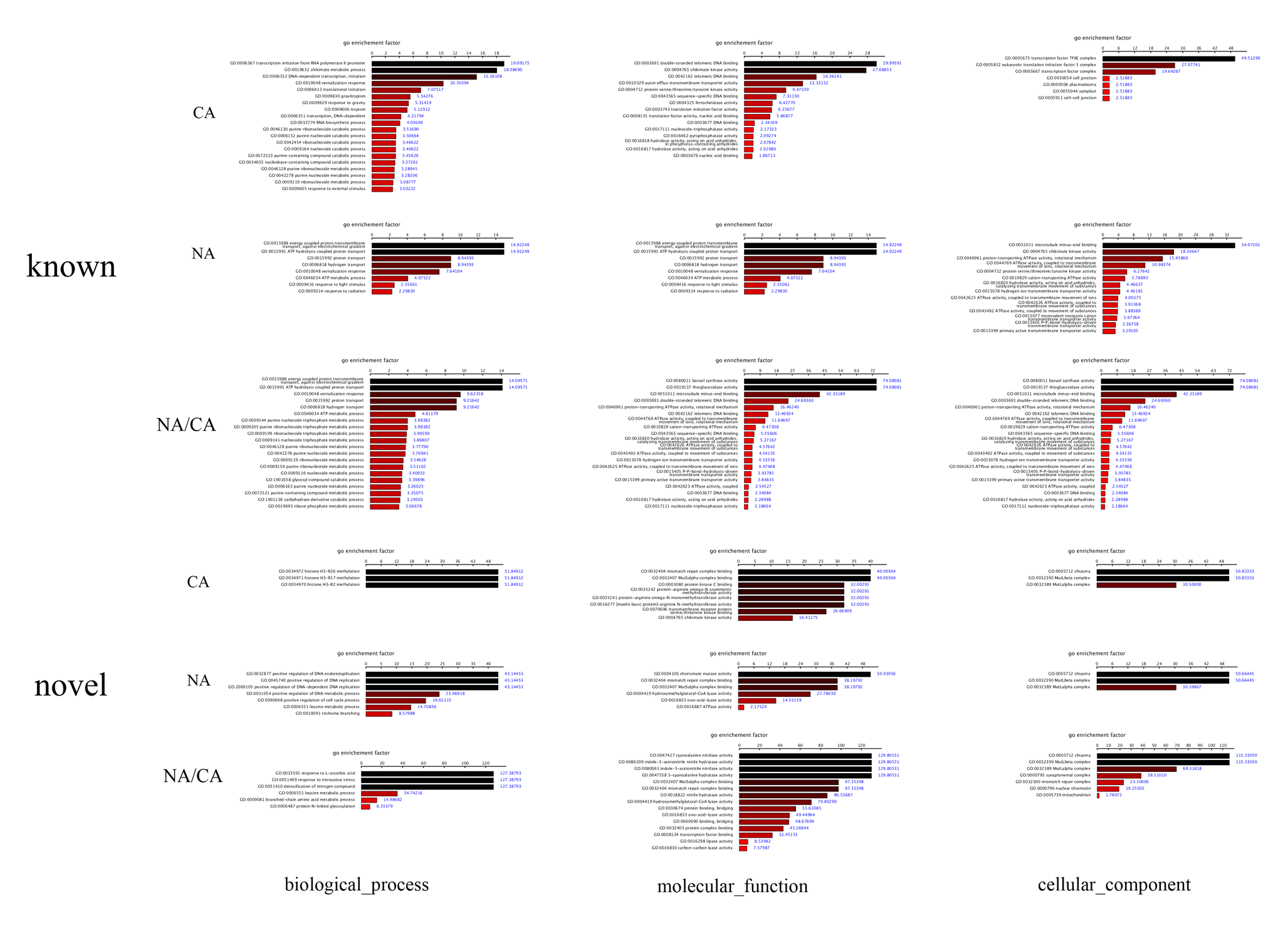

Supplement: Figure S2 — Enrichment analysis of miRNA targets. [file Image2.TIF]

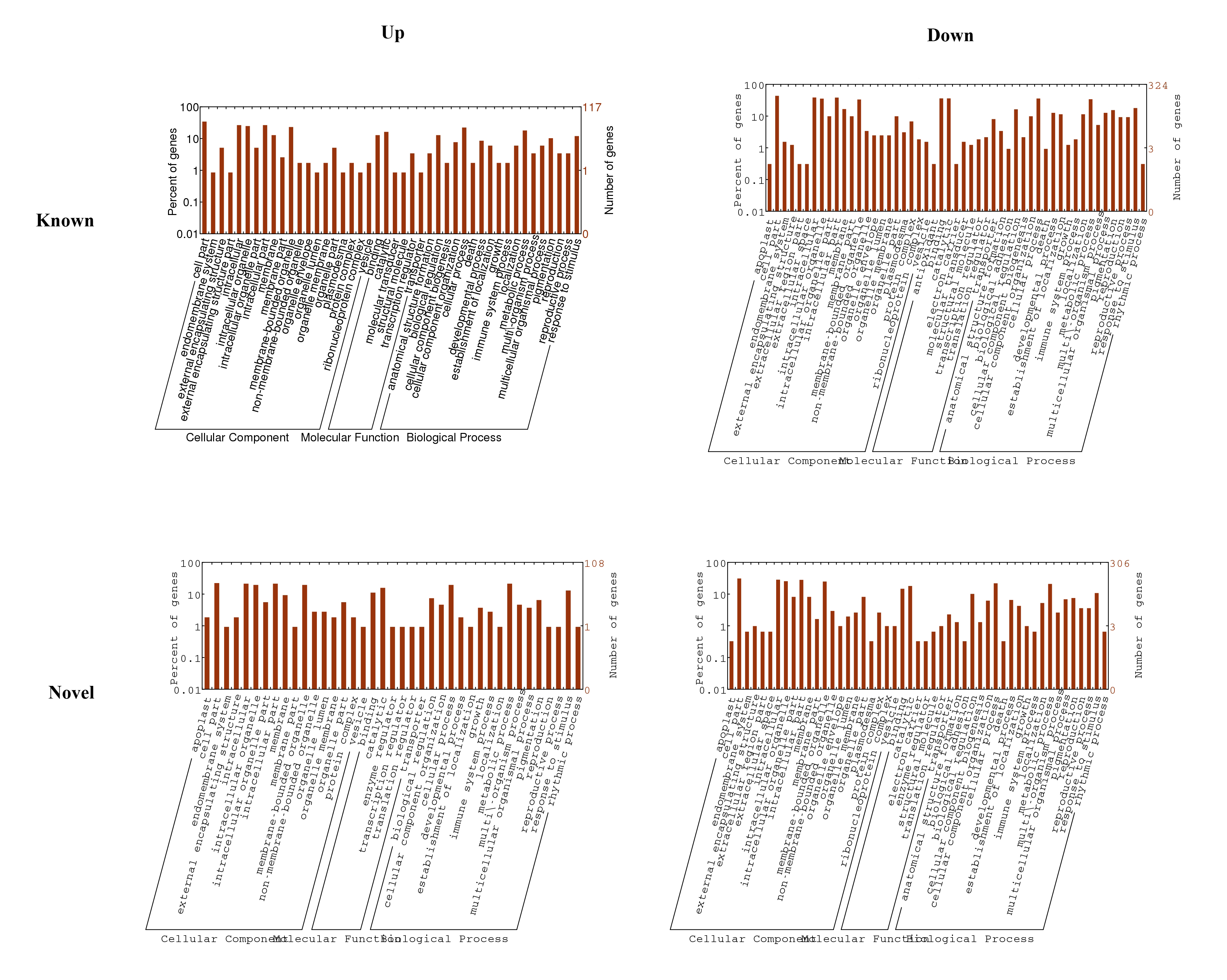

Supplement: Figure S3 — GO analysis of up- and downregulated targets. [file Image3.TIF]
