# Supplementary material for: High-throughput sequencing reveals miRNA effects on the primary and secondary production properties in long-term subcultured Taxus cells
Source: Front Plant Sci. 2015 Aug 6;6:604. doi: 10.3389/fpls.2015.00604 (PMC4527571; doi:10.3389/fpls.2015.00604)
Supplement: Table S1 — Quality statistics of sequencing tags of miRNAs. [file Table1.DOC]

**Table S1 Quality statistics of sequencing tags of miRNA**

|  | CA | NA |
| --- | --- | --- |
| total_reads | 19,998,242 | 19,686,076 |
| high_quality | 19,843,361 | 19,558,835 |
| 3'adapter_null | 13,084 | 16,080 |
| insert_null | 10,954 | 10,011 |
| 5'adapter_contaminants | 172,820 | 176,699 |
| smaller_than_18nt | 145,466 | 142,846 |
| polyA | 718 | 581 |
| clean_reads | 19,500,319 | 19,212,618 |
